# Building Unconventional Magnetic Phases on Graphene by H Atom Manipulation: From Altermagnets to Lieb Ferrimagnets

**DOI:** 10.1021/acs.nanolett.5c02091

**Published:** 2025-07-17

**Authors:** Beatriz Viña-Bausá, Manuel Antonio García-Blázquez, Simran Chourasia, Roberto Carrasco, Diego Expósito, Iván Brihuega, Juan José Palacios

**Affiliations:** † Departamento de Física de la Materia Condensada, Universidad Autónoma de Madrid, E-28049 Madrid, Spain; ‡ Condensed Matter Physics Center (IFIMAC), Universidad Autónoma de Madrid, E-28049 Madrid, Spain; ¶ Instituto Nicolás Cabrera (INC), Universidad Autónoma de Madrid, E-28049 Madrid, Spain

**Keywords:** altermagnetism, compensated ferrimagnetism, atomic manipulation, graphene spintronics, scanning tunneling microscopy (STM)

## Abstract

Engineering all magnetic phases within a single material platform would mark a significant milestone in materials science, simplifying device fabrication by eliminating the need for the integration of different materials. Here, we demonstrate that graphene can host all nonrelativistic magnetic phases—diamagnetism, paramagnetism, ferromagnetism, antiferromagnetism, ferrimagnetism, altermagnetism, and fully compensated ferrimagnetism—using single H atoms as building blocks. Their magnetic character is confirmed by density functional theory and mean-field Hubbard calculations. Notably, altermagnetism can be realized, exhibiting directionally spin-split bands coexisting with zero net magnetization due to spatial symmetries. Furthermore, fully compensated ferrimagnets can be created, lacking these symmetries and presenting unrestricted spin-splitting, with vanishing net magnetization protected by Lieb’s theorem. We take this idea to the laboratory and, through the precise manipulation of H atoms by scanning tunneling microscopy, experimentally create isolated unit cells of all magnetic phases. These findings open the door to the bottom-up design of magnetic phases via symmetry selection.

## Graphical abstract

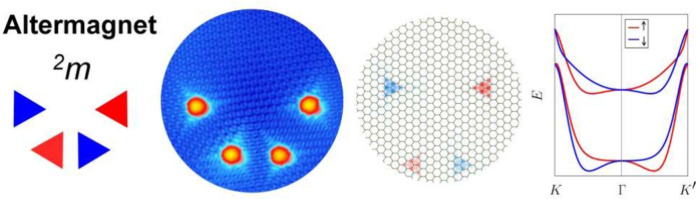

A new class of magnetic materials, merging the advantages of both ferromagnetic and antiferromagnetic systems, holds the potential to revolutionize next-generation spintronic devices. These materials, notably those known as altermagnets but also a variant of ferrimagnets, are characterized by zero net magnetization, similar to antiferromagnets yet retaining the spin-split electronic band structures characteristic of ferromagnets. This unique combination minimizes issues related to stray magnetic fields, facilitating miniaturization and device integration while allowing spin-polarized electronic transport without the need for external magnetic fields or relativistic effects. Moreover, these materials offer directionally dependent electronic properties and tunable magnetic features and hold significant potential for faster operational speeds, lower power consumption, and enhanced thermal transport.^1–5^

The study of altermagnets, in particular, is rapidly progressing.^1–4,6,7^ In 2024, driven by recent theoretical and computational efforts, different crystalline systems, including MnTe,^8–10^ RuO_2_,^11^ and CrSb,^12^ were reported as the first experimental realizations of altermagnetism. On the other hand, the combination of zero net magnetization and spin-split bands, present in altermagnets, is shared by another class of magnetic systems—the fully compensated ferrimagnets. While in altermagnets zero net magnetization is protected by space-time symmetries, in compensated ferrimagnets it is achieved by fine-tuning the composition or structure with external manipulations.^13–19^ Until now, theoretical and experimental efforts have primarily focused on a materials-based approach—identifying or synthesizing compounds that inherently possess the desired properties, hosted by a crystalline lattice with a suitable symmetry group upon magnetization.

In this work, following spin group symmetry principles, we propose an alternative bottom-up strategy to build unconventional compensated magnetic states, specifically altermagnets and compensated ferrimagnets. We have selected hydrogen (H) atoms on graphene as our working platform.^20^ This system combines several inherent properties that, put together, allow for the formation of all magnetic phases, conventional and unconventional, on a single material. In brief, our proposed platform offers (a) zero net magnetization, guaranteed by Lieb’s theorem,^21^ when there is an equal concentration of H atoms on both graphene sublattices, (b) manipulation capability of magnetic moments with atomic scale control, (c) an anisotropic shape of the H-induced magnetic moment, and (d) a long-range exchange interaction of both ferromagnetic and antiferromagnetic nature. With these ingredients at hand, we show how all possible collinear, nonrelativistic magnetic phases could be achieved by adjusting the arrangement of up to 4H atoms that form the unit cell. Additionally, a highly symmetrical altermagnetic phase can be realized with some particular arrangements involving 6H atoms.

We also take a first, preliminary step toward the experimental verification of our theoretical predictions through the construction of individual unit cells. Since spatial and spin symmetries determine the magnetic phases that can emerge, the practical capability to visualize and control them at the atomic scale is a key requirement of our bottom-up strategy. Scanning tunneling microscopy (STM) manipulation^22^ provides such functionality, allowing the strategic positioning of individual atoms on surfaces,^23–25^ thus enabling the construction of configurations with the appropriate symmetry. Atomically resolved STM topographic images facilitate, in turn, the analysis of the existing spatial and, indirectly, spin symmetries.

The adsorption of a single H atom on graphene induces a magnetic moment without the need of external, intrinsically magnetic, elements.^26,27^ The covalent bond between the hydrogenic s orbital and the carbon p_*z*_ (out-of-plane) orbital effectively removes an electron from the corresponding graphene sublattice, leaving an unpaired electron and therefore generating a magnetic moment localized mostly in the complementary graphene sublattice. The spin cloud extends over several nanometers, exhibiting an anisotropic, triangular shape. The induced magnetic moments couple ferromagnetically (antiferromagnetically) for H atoms adsorbed on the same (complementary) sublattice and are essentially collinear, aided by the low strength of spin orbit coupling (SOC) in graphene. More generally, as follows from (the second) Lieb’s theorem for half-filled bipartite lattices,^21^ in a system with *N*_*A*_ and *N*_*B*_ H atoms chemisorbed on each graphene sublattice, the total spin in the ground state must be *S* = |*N*_*A*_ – *N*_*B*_|/2. By individually manipulating H atoms with a STM tip, one can achieve a desired distribution of these atoms across both sublattices,^20^ which enables selectively tuning the material’s magnetization.

Traditionally, magnetic systems have been described using (relativistic) magnetic groups, where symmetry operations act simultaneously on spin and real space.^28–30^ In systems with negligible SOC (compared to the nonrelativistic magnetization) additional symmetry operations appear, since those acting on the spin degrees of freedom decouple from the electronic coordinates. The corresponding framework is that of (nonrelativistic) spin groups,^2,3,31,32^ where symmetry transformations are applied independently on spin and real space. We thereby employ the notation [*A*∥*B*] for a general spin group operation, where *A* (*B*) acts exclusively in real (spin) space. The use of spin groups enables the prediction of features that would only be approximate from magnetic groups, in particular, the centrosymmetry of the band structure (*ε*_σ, ***k***_ = *ε*_σ,–***k***_ for σ = *↑* and *↓*) irrespective of the spatial symmetries of the system as long as the magnetization is collinear.

In simple terms, this symmetry-based procedure constructs different magnetic phases by identifying spatial transformations that return a given system to its initial state after a spin-flip operation. In terms of spin groups, the global spin-flip can generally be achieved with [e∥P] , where *e* and P denote the identity and inversion operation, which must be antiunitary (i.e., involve complex conjugation) and can thus be identified with time-reversal T . Therefore, we employ the notation [e∥P] or T interchangeably. For uncompensated systems, such as ferromagnets and general ferrimagnets, after a spin-flip operation, there is no real-space transformation that restores the original configuration. In compensated systems, however, different spatial symmetries define the existing magnetic phases. In antiferromagnets, characterized by a global spin degeneracy (or Kramer’s degeneracy) the defining symmetry is, [P∥P] (inversion in both real and spin spaces) or, in the case of 2D systems, alternatively $\left[C_{2,z}∥P\right]$ (*C*_2,*z*_ being a 2-fold rotation axis perpendicular to the lattice). In the novel altermagnetic phase,^1^ the system is returned to its initial state by a different spatial operation following the spin-flip, such that Kramer’s degeneracy is lifted. Finally, in fully compensated ferrimagnets, after performing a spin-flip operation, there is no real-space symmetry restoring the initial configuration.

As it was previously established,^20,26^ and we here summarize in Figure 1, all conventional magnetic phases can be formed with up to 3H atoms on graphene: diamagnetism in the bare carbon lattice (0H), paramagnetism with 1H atom, ferromagnetism with 2H atoms on the same sublattice, antiferromagnetism with 2H atoms on complementary sublattices (as long as the separation between them is larger than ≳1.5 nm^33^), and ferrimagnetism with 2H atoms on one sublattice and 1H atom on the complementary sublattice. This classification is not altered by introducing translational symmetry elements, namely, by considering periodic arrangements of these structures. A comparison with density functional theory (DFT) calculations reveals a clear correlation between the STM topography and the anisotropic, triangular-shaped distribution of the magnetic moment induced by a single H atom; see Figure 1 and Figure 2a. For H atoms located on different graphene sublattices, the triangular magnetic distributions are predominantly of opposite spins and point in opposite directions, enabling the identification of the relative spin orientations in the experiment: parallel for triangles oriented in the same direction and antiparallel for those oriented oppositely.

**Figure 1:**
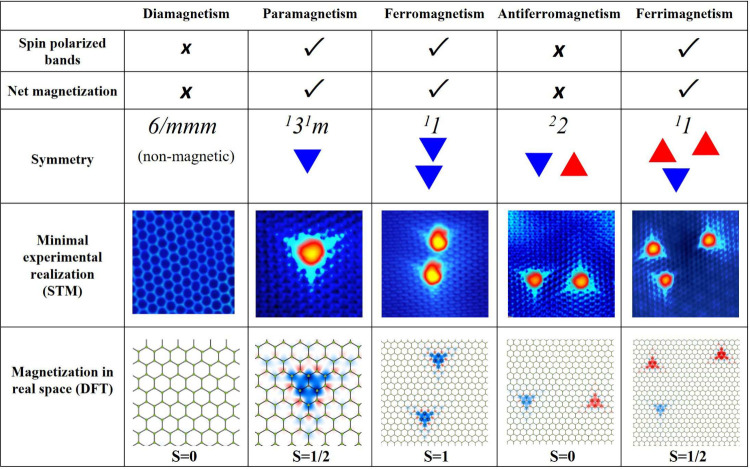
Building conventional magnetic phases using H atoms on graphene. All possible nonrelativistic, conventional magnetic phases can be realized on graphene by incorporating up to 3H atoms. Point groups are indicated, in the spin group notation, for magnetic configurations presenting nontrivial symmetries. The two bottom rows show STM images and DFT-calculated magnetizations for each magnetic phase, clearly revealing that STM images encode the triangular magnetic orbital shape and the graphene sublattice adsorption site determining spin orientation. Representing each H atom as red or blue triangle (indicating opposite spin orientations) highlights the 3-fold anisotropy of the induced magnetic state. This symbolic approach enables the independent application of symmetry operations in both real and spin space, aiding in the design and identification of specific magnetic phases. STM image parameters by columns: (80 mV, 0.1 nA, 2.5 × 2.5 nm^2^); (50 mV, 0.1 nA, 4.7 × 4.7 nm^2^); (30 mV, 0.1 nA, 6.2 × 6.2 nm^2^); (40 mV, 0.1 nA, 7.5 × 7.5 nm^2^); (4 mV, 0.05 nA, 7.3 × 7.3 nm^2^).

**Figure 2:**
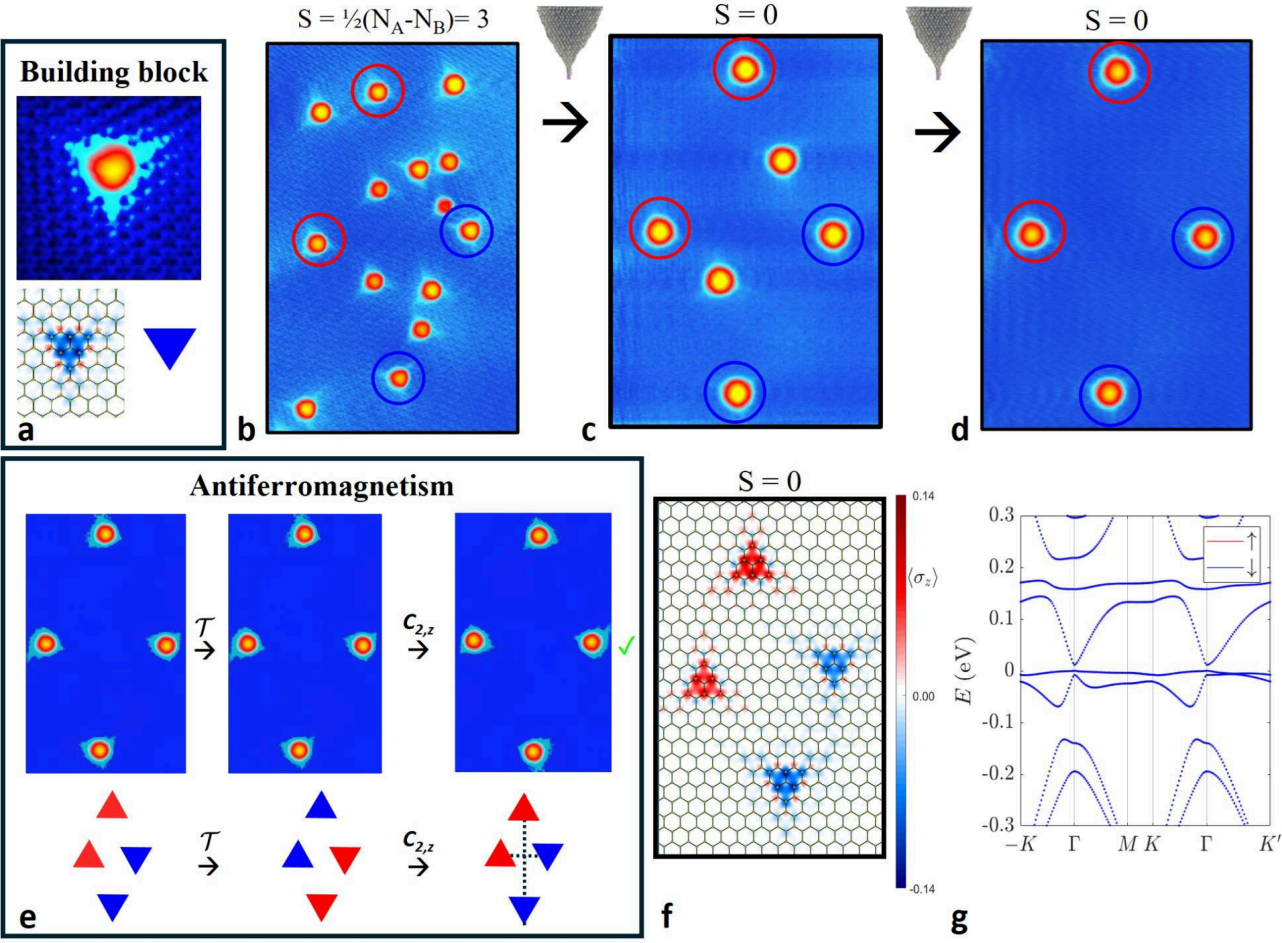
Building magnetic phases atom by atom. The antiferromagnetic case. (a) STM image (top) and DFT calculated magnetization (bottom left) of a single H atom on graphene. Schematized as a triangle (bottom right), it is proposed as a *S* = 1/2 building block for engineering magnetic phases on graphene. (b–d) Sequence of STM images of the same graphene region, where selected H atoms are subsequently removed to construct a 4H parallelogram (to construct a *C*_2,*z*_ symmetric structure corresponding to an antiferromagnet). (e) Symmetry operations in spin and real space applied to both the experimental STM image and its symbolic representation. After the time-reversal operation, the 2-fold rotation brings the system back to its initial state. (f–g) DFT-calculated magnetization and energy bands of a rescaled configuration showing a total spin *S* = 0 and fully spin degenerated energy bands. STM image parameters: (b) 40 mV, 0.1 nA, 15.2 × 24 nm^2^, (c–e) 80 mV, 0.1 nA, 15.2 × 22 nm^2^.

In order to realize unconventional magnetism, it is necessary to consider more H atoms in the unit cell. In this work, we mainly focus on 4H arrangements, which represent the minimal configurations necessary to form both altermagnetic and compensated ferrimagnetic phases. We show their magnetic properties and how we experimentally constructed the three magnetically compensated structures that can be formed in graphene: antiferromagnets, altermagnets, and Lieb (compensated) ferrimagnets.

As explained in the discussion, with 4H atoms, the antiferromagnetic case must be realized with a parallelogram geometry. In Figure 2b–d, we show a sequence of STM images measured on the same graphene region, illustrating the manipulation of atomic hydrogen to construct such a 4H arrangement. Initially, multiple hydrogen atoms are gathered from the graphene surface onto the STM tip apex; see the Methods for details. As shown in Figure 2b, we subsequently deposit the hydrogen atoms onto a selected region of the graphene surface. While atomic-scale precision is not achieved during the deposition step, we control the final structure by selectively removing individual H atoms to achieve the desired configuration and symmetry. In this case, an antiferromagnetic parallelogram can be built by removing all H atoms except those marked with circles, where the red and blue colors denote the adsorption sublattices, corresponding to opposite spin orientations. Figure 2c shows the same graphene region after we selectively removed 8H atoms to create a magnetically compensated configuration with an equal number of hydrogen atoms on each sublattice (*N*_*A*_ = *N*_*B*_ = 3). As guaranteed by Lieb’s theorem, the region has a total vanishing net magnetization, while still lacking $\left[C_{2,z}∥P\right]$ symmetry. In the final step, shown in Figure 2d, the remaining 2H atoms were removed to create the desired 4H parallelogram.

Spatial symmetries are encoded in the local density of states measured in the topographic STM images, combining the contributions of both spin states. It is then easy to verify the symmetries from these measurements, recalling the well-established association: equal sublattice ↔ equal magnetic moment orientation. Time-reversal (or spin-flip) operations T=[e∥P] do not affect the appearance of the STM image, as they operate solely within spin space. The application of the *C*_2,*z*_ rotation to the STM image in this case restores the system to its initial configuration, see Figure 2e, and the product $\left[C_{2,z}∥P\right]$ is therefore a symmetry of the system. Such symmetry enforces the global spin degeneracy of the bands in nonrelativistic 2D systems with collinear magnetism. This is confirmed by DFT calculations; see Figure 2f–g, displaying the characteristic zero net magnetic moment and fully degenerate energy bands. These calculations were conducted using a supercell approach to reveal band structures; in particular rescaling to a smaller size of 24 × 24 (1156 atoms) due to computational constraints, while keeping the proportions of the experimental geometry. Mean-field Hubbard tight-binding calculations are also presented in Figure S1.

In order to form an altermagnetic state, it is necessary to remove the $\left[C_{2,z}∥P\right]$ symmetry while still maintaining a nontrivial spin group. The complete discussion can be found below, with the main conclusion being that there are two spin groups compatible with hydrogenated graphene. These are ^2^ *m*, with a mirror plane perpendicular to the lattice (vertical) and paired with spin inversion, and ^1^3^2^ *m*, with a 3-fold rotational symmetry not paired with spin inversion and three vertical planes paired with spin inversion. As shown in Figure 3a, we used STM manipulation to create a 4H arrangement exhibiting the ^2^ *m* spin group. As observed, applying mirror symmetry to the STM image after time-reversal restores the initial configuration. In contrast to the antiferromagnetic arrangement, the system lacks $\left[C_{2,z}∥P\right]$ symmetry (see Figures S2 and S3). To validate our conjecture and confirm that this system exhibits the expected properties for an altermagnet, we conducted mean-field Hubbard tight-binding and DFT calculations, respectively, included in Figure 3b–d and Figure S4. As predicted by Lieb’s theorem, the calculations show zero net magnetization for the system. Importantly, analysis in reciprocal space reveals spin-polarized energy bands along specific directions, as expected for an altermagnet. This behavior is consistently observed across various configurations of H atoms with equivalent symmetry properties; see Figures S4 (DFT) and S5 (tight-binding). As revealed in Figure 3d, the spin texture of the bands along the Brillouin zone exhibits a d-wave symmetry, with the centrosymmetry of the texture resulting from the absence of an SOC.

**Figure 3:**
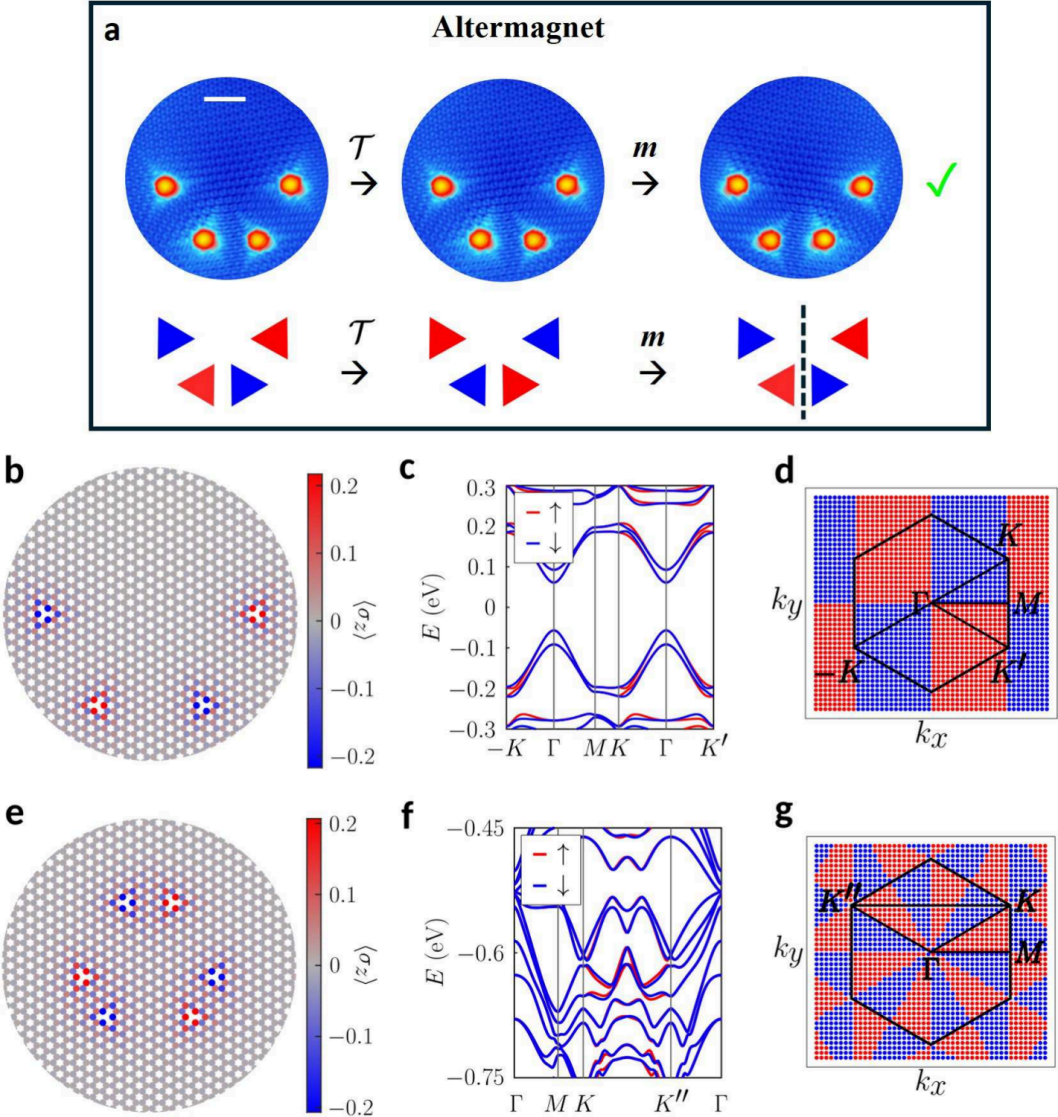
Hydrogenated graphene as an altermagnet. (a) Top: STM image (120 mV, 0.1 nA, scale bar = 2 nm) and symmetry transformations of the minimal experimental realization of an altermagnet, built using the STM tip, by arranging 4H on alternating graphene sublattices with a vertical mirror symmetry (^2^ *m* spin group). Bottom: Triangle schematics and symmetry transformations of the configuration. By applying T (inversion in spin space) and a vertical mirror symmetry in real space we recover the original configuration. (b–c) Atomic magnetization and energy bands of a rescaled configuration corresponding to (a), calculated using a mean-field Hubbard model. (d) Spin texture of the highest occupied valence band in the (b–c) configuration across reciprocal space, where the hexagon delimits the Brillouin zone and red (blue) color represents spin *↑* (*↓*). The texture corresponds to a *d* wave. (e–f) Atomic magnetization and energy bands corresponding to a different configuration with spin group ^1^3^2^ *m*, calculated from a mean-field Hubbard model. (g) Spin texture of a band in the (e–f) configuration across reciprocal space, exhibiting an *i* wave texture that is realized in 2D due to the absence of spin–orbit coupling. For the 6H configuration in (e), the bands show more splitting as we go away from the Fermi level.

The remaining altermagnetic phase, with the ^1^3^2^ *m* spin group as predicted by the symmetry analysis, requiring a configuration with at least 6H atoms, is here demonstrated by the mean-field Hubbard tight-binding calculation (see Figure 3e–g). The spin texture exhibits an *i* wave symmetry due to the reduced dimensionality, as the same spin group would lead to a *g* wave in 3D.^2^

4H configurations can also exhibit zero net magnetization with fully spin-polarized bands (notice that this is impossible with fewer H atoms). We refer to these configurations as “Lieb ferrimagnets”, drawing an analogy to existing fully compensated ferrimagnets.^34^ The key distinction lies in the fact that, in this case, the compensation of both spin states (resulting in a vanishing total magnetization) is enforced by Lieb’s theorem rather than by a specific magnetic space group lacking space-time inversion symmetry. Additionally, the spatial symmetry in these configurations is broken by individual H atoms being displaced from their symmetry positions in real space, as expected from random H arrangements, rather than by fine-tuned compositions or external conditions as in traditional ferrimagnets.^13–19^ Figure 4 illustrates an experimental realization of the Lieb ferrimagnetic phase, achieved with 4H atoms slightly displaced from an altermagnetic configuration. In this case, after a spin-flip, the system does not revert to its original configuration regardless of the subsequent spatial symmetry transformation applied to the experimental STM topography; see Figure 4a. Our DFT and mean-field Hubbard calculations confirm that the system exhibits zero net magnetization and fully spin-polarized energy bands; see Figure 4b–c and Figure S6, respectively. This behavior has been consistently verified across numerous broken-symmetry, fully compensated configurations (Figure S7).

**Figure 4:**
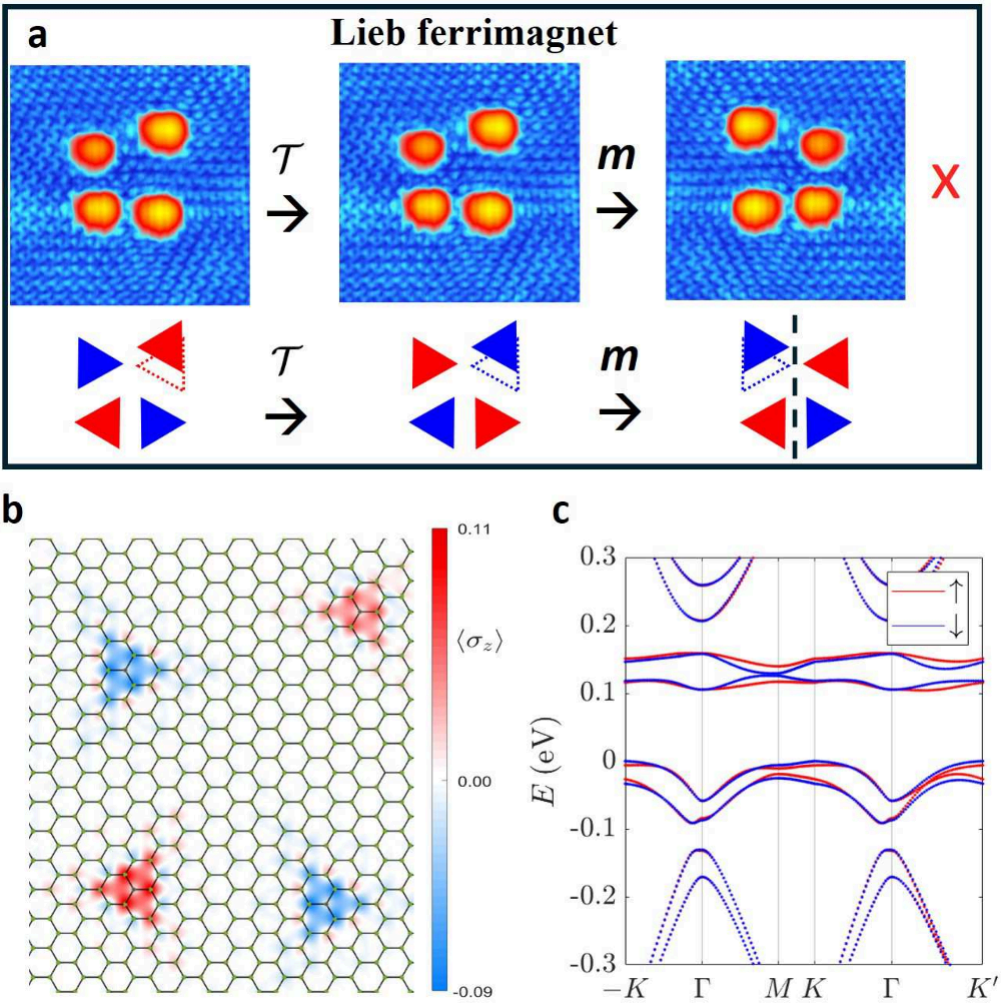
Lieb ferrimagnet. (a) Top, STM image (50 mV, 0.1 nA, 7 × 7 nm^2^) and symmetry transformations applied to the minimal experimental realization of a Lieb ferrimagnet. Bottom, triangle schematics and symmetry transformations. Dashed triangles highlight how crystal symmetry is broken. After T (inversion in spin space), there is no real-space operation that recovers the initial configuration. (b) DFT magnetization of the experimental broken symmetry configuration, with zero net magnetization. (c) Spin resolved electronic band structure of the configuration shown in (b). The spin splitting is unrestricted due to the absence of any symmetry, and the perfect magnetization compensation is enforced by Lieb’s theorem.

Importantly, as shown in Figure S6d–e, our mean-field tight-binding calculations demonstrate that the spin polarization of the bands in this new magnetic phase is robust against small levels of doping. This enables the long-sought ability to tune the spin polarization of currents through external electrostatic doping. In Figure S8, we summarize the three possible magnetically compensated structures that can be created in graphene with 4H atoms.

In 2D materials, Kramer’s degeneracy is generally enforced by the space-time inversion symmetry $\left[C_{2,z}∥P\right]$ . Upon the adsorption of an arbitrary number of H atoms on the graphene lattice, *C*_2,*z*_ is generally removed except in some particular cases. Specifically, for 2H atoms $\left[C_{2,z}∥P\right]$ is only preserved if they are adsorbed on different sublattices, and for 4H atoms only if they form a parallelogram (with the rotation axis containing its centroid), with 2H in each sublattice. Therefore, only the electronic band structure of these specific configurations is Kramer’s degenerate while the remaining systems exhibit a certain spin-splitting. Concomitantly, by Lieb’s theorem^21^ any configuration with an evenly distributed number of H atoms among both sublattices will present a vanishing total magnetic moment in the ground state. Therefore, a graphene lattice with an even number of adsorbed H atoms equal to or greater than 4, distributed evenly among both sublattices, will in general exhibit a ground state with perfectly compensated, collinear magnetism, and spin-split bands (if the fundamental unit cell is repeated). The only exceptions are H arrangements with a center of inversion, namely, those for which the H atoms form a zonogon.

While random, evenly distributed arrangement of H atoms will most probably correspond to a Lieb ferrimagnet, it is however possible to engineer altermagnetic states by placing the H atoms in symmetry-related sites, in reminiscence of the antiferromagnetic construction with 4 H shown in Figure 2b–d. Altermagnets are essentially classified according to their spin point group. There are 37 possibilities in 3D systems (not all of them compatible with lower dimensionalities, and no more options appear in 2D), with the 10 ones that contain inversion named Laue groups. The incoming discussion can be followed with the aid of, for example, Table S.I of ref 2 (see the Supporting Information therein). First we exclude all cubic and tetragonal groups, which are not compatible with the hexagonal lattice of graphene. Furthermore, we disregard all groups that include the inversion of *z*, which is not an essential limitation in any case since these operations do not affect reciprocal space and there always exists a partner group that excludes such operations (hence the case of vacancies instead of H atoms is qualitatively analogous). From the observation that there is a one-to-one correspondence between sublattice of adsorption and spin orientation, it follows that any spatial operation that permutes (does not permute) the sublattices would necessarily have to be paired (not be paired) with the spin inversion operation in order to be a symmetry of the hydrogenated graphene system. The remaining spatial operations that permute the sublattices are *C*_2,*z*_, *C*_6,*z*_, and (vertical) mirror planes bisecting the carbon bonds, while the operations that do not permute the sublattices are *C*_3,*z*_ and the (vertical) mirror planes passing through carbon atoms. However, from these possibilities it is still necessary to discard the groups containing *C*_2,*z*_ in order to avoid Kramer’s degeneracy in the spin group. Combining all of these arguments, we conclude that the only possible spin groups that are compatible with our platform are ^2^ *m*, with the mirror plane perpendicular to the lattice, and ^1^3^2^ *m*. Both are non-Laue spin groups, respectively presenting *d* and *g* wave parities or textures in 3D reciprocal space;^2^ however, the latter is further subdivided within the planar Brillouin zone by the nonrelativistic centrosymmetry resulting in an *i* wave symmetry. It is clear that the former group can be realized with any even number of H atoms excluding 2 (which always preserves $\left[C_{2,z}∥P\right]$ , assuming opposite sublattices), while the latter requires a multiple of 6. Both of these distinct altermagnetic configurations are shown in Figure 3.

We note that the previous reasoning can equally be applied to both crystalline and finite cluster structures, since the spin point groups are the same. Certain qualitative properties of the cluster structures, which are definitely more straightforward to build, are therefore expected to be shared with the crystalline ones, for which the altermagnetic description is clear in terms of the band structure. In particular, following the analysis in ref 35 for the spin polarization of the transmitted current in transport setups through clusters, here adapted to the context of spin groups (whereby the spin inversion can be realized without complex conjugation), we expect to find a finite spin polarization for clusters in a Lieb-ferrimagnetic configuration. For clusters in the altermagnetic phase, the spin polarization vector should, in contrast, depend on the direction of the current with respect to the cluster; in particular we expect it to vanish when the current is aligned with a symmetry plane due to the [*m*∥*C*_2,⊥_] symmetry (something which, furthermore, suppresses a possible magneto-conductance signal in the nonlinear regime^36^). This, indeed, constitutes a directionally dependent spin response for the cluster derived from the altermagnetic phase, as expected with the usual depiction in terms of band structures but here ultimately arising from the spin point group. Furthermore, the local spin polarization could be measured with a spin-polarized STM.

In conclusion, we have introduced a bottom-up strategy to engineer magnetic structures that simultaneously exhibit perfect spin compensation and lifted spin degeneracy using graphene and individual H atoms as building blocks. Owing to Lieb’s theorem, this approach can be scaled up to a large number of hydrogen atoms while maintaining zero net magnetization, provided both graphene sublattices are equally populated. We have shown that configurations with (spatial) vertical mirror symmetry serve as fundamental units of altermagnets, whereas those lacking such symmetry yield Lieb-ferrimagnets. In both scenarios, $\left[C_{2,z}∥P\right]$ symmetry is broken, leading to a spin-polarized band structure coexisting with zero net magnetization. By enabling direct visualization and manipulation of spatial and spin symmetries at the atomic scale, our technique opens new avenues for the targeted design of magnetic phases. This concept can be extended to other substrates and atomic species or applied through self-assembly techniques to realize altermagnets from molecular arrays on surfaces. Additionally, the required symmetry conditions may be tailored via strain or twisting in two-dimensional materials, facilitating the creation of both altermagnets and fully spin-split, compensated ferrimagnets.

## Supplementary Material



## Notes

Glossary, sample preparation and STM measurements, atomic H manipulation, density functional theory results, mean-field Hubbard model, supporting figures (PDF)
