# Supplementary material for: Building Unconventional Magnetic Phases on Graphene by H Atom Manipulation: From Altermagnets to Lieb Ferrimagnets
Source: Nano Lett. 2025 Jul 17;25(30):11554–61. doi: 10.1021/acs.nanolett.5c02091 (PMC12314891; doi:10.1021/acs.nanolett.5c02091)
Supplement: Supplementary file 1 [file nl5c02091_si_001.pdf]

Building unconventional magnetic phases on graphene by H atom manipulation: From altermagnets to Lieb ferrimagnets - Supporting Information

Beatriz Viña-Bausá,[†] Manuel Antonio García-Blázquez,[†] Simran Chourasia,[†] Roberto Carrasco,[†] Diego Expósito,[†] Iván Brihuega,[*,†,‡,¶] and Juan José Palacios[†,‡,¶]

†*Departamento de Física de la Materia Condensada, Universidad Autónoma de Madrid, E-28049 Madrid, Spain*

‡*Condensed Matter Physics Center (IFIMAC), Universidad Autónoma de Madrid, E-28049 Madrid, Spain.*

¶*Instituto Nicolás Cabrera (INC), Universidad Autónoma de Madrid, E-28049 Madrid, Spain.*

E-mail: ivan.brihuega@uam.es

Glossary

Symmetry Transformations:

e	identity
$\mathcal{P}$	inversion
$\mathcal{T}$	time-reversal (inversion $\mathcal{P}$ in spin-space)
$C_{n,z}$	$n-$fold rotation ($2\pi/n$ rad) along the $z-$axis ($\perp$ lattice)
m	mirror reflection

Notation for operations in spin groups:

$$[A \parallel B]$$

A acts exclusively on real-space.

B acts exclusively on spin-space. Both A, B are members of the orthogonal group $O(3)$. If B is not a member of $SO(3)$ (i.e. it does not preserve orientations, such as $\mathcal{P}$) then it is accompanied by complex conjugation in the formalism of spin groups.

Hermann-Mauguin notation for spin point groups compatible with hydrogenated graphene: Derived from the Hermann-Mauguin notation of ordinary point groups by adding a superscript B to the left of each symbol. For collinear magnetic states, there are essentially two options for B. If $B = 1$ ($\neq 1$), the corresponding spatial operation or set of operations preserves (inverts, respectively) spin. We choose $B = 1, 2$ for consistency with the literature (e.g. Ref.[1]), which corresponds to a C_2 in spin space perpendicular to the collinear magnetization, but note that any operation in spin-space that inverts such direction (e.g. $\mathcal{P}$) equally yields a symmetry of the spin group. The relevant spin groups for the present work are listed in the following table. In the left column, we show the spin groups in Hermann-Maugin notation, and in the right column the corresponding group elements.

The following spin groups correspond to the different H configurations discussed in this work.

$^{1}1$	$[e \parallel e]$
$^{1}3^{1}m$	$[e \parallel e], [C_{3,z} \parallel e], [C_{3,z}^{2} \parallel e], [m_1 \parallel e], [m_2 \parallel e], [m_3 \parallel e]$
$^{2}2$	$[e \parallel e], [C_{2,z} \parallel \mathcal{P}]$
^{2}m	$[e \parallel e], [m \parallel \mathcal{P}]$
$^{1}3^{2}m$	$[e \parallel e], [C_{3,z} \parallel e], [C_{3,z}^{2} \parallel e], [m_1 \parallel \mathcal{P}], [m_2 \parallel \mathcal{P}], [m_3 \parallel \mathcal{P}]$

Example:

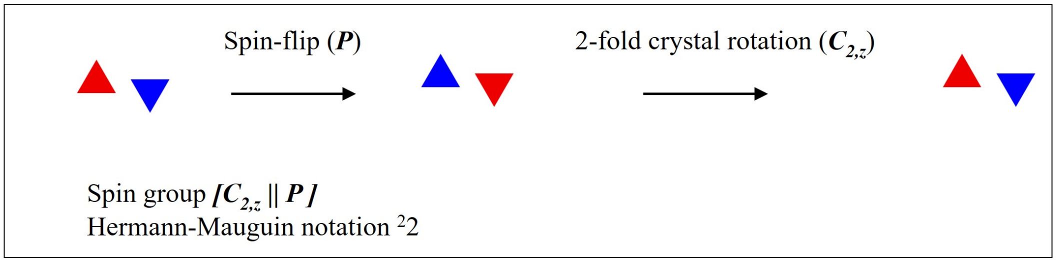

In the above example, we apply a spin-flip, which interchanges the colors red and blue, followed by a (180°) real-space rotation. This combined operation restores the system to its initial configuration.

Sample preparation and STM measurements

Multilayer graphene is grown epitaxially in ultra-high vacuum (UHV) conditions by thermal decomposition of a 6H-SiC(000-1) sample.[2] In this system, due to the rotational disorder the upmost graphene layer is neutral and electronically decoupled, behaving essentially as free-standing graphene.[3]

We deposit atomic H on graphene by thermal dissociation of a beam of H_2 using a hot W filament.[4,5] H_2 pressure is regulated by a leak valve and fixed to $4 \cdot 10^{-7}$ Torr as measured in the preparation chamber. The filament is held at 1900K for 6 minutes with the sample placed 10 cm away at room temperature. During the whole process—imaging pristine graphene sample => depositing H atoms on it => and imaging it back—the sample

was maintained in the same UHV system.

All experiments were performed on a homemade STM at a temperature of 4K. The STM data was acquired and processed using the WSxM software.[6]

Atomic H manipulation

Different H-atom arrangements are created by atomic manipulation using the STM tip. Selected graphene regions are "cleaned" by removing, and thus collecting, all H atoms with the tip. This collection can be carried out either atom-by-atom—by contacting each H atom individually at low voltages—or collectively, by rapidly scanning the surface at low bias voltages and high currents, (5-100mV and 5-10nA). The collected H atoms are deposited on a selected graphene region by applying negative sample voltages pulses (up to -9V). A slightly variable number of H atoms is thus deposited with nm scale precision. To create the intended design, with the desired total magnetization and symmetry, excess H atoms are removed one by one, approaching the STM tip (1-2 Å) to their adsorption site, leaving only the desired pattern. At present, the deposition step is subject to some incertitude, determined by the specific tip apex termination. However, the selective removal of single H atoms works with essentially a 100% efficiency, enabling the precise construction of the selected symmetries.

Density functional theory results

The self-consistent electronic Hamiltonian was obtained employing the massively parallel version of the `CRYSTAL` code,[7] in all cases with the standard PBE exchange-correlation functional.[8] We employed Gaussian basis sets consisting of a reduced pob-DZVP-rev2[9] (removing the $d-$type functions) for carbon, suitable for the description of a bare graphene lattice, and an unmodified pob-TZVP[10] for hydrogen. A single geometry optimization was performed with one H atom in a 12×12 supercell, restricted to the carbon atom in the adsorption

site, its 3 nearest neighbors and the H atom; resulting in an attraction of the carbon cluster towards the H atom and in particular a C-H bond length of 1.13Å, with the central carbon lifted 0.38Å above the lattice. These displacements were individually applied for each H region in subsequent calculations, which were carried out in a 24×24 supercell to minimize spurious interactions within the computational capabilities. Experimental configurations are rescaled to fit the supercell size, keeping the proportions of the shape defined by the H vertices as unaltered as possible. A threshold of 10^{-10} for overlap and penetration of Hartree integrals (see Ref.[11]) was employed, in conjunction with an uniform sampling of 6×6 points in the Brillouin zone of the supercell. An initial magnetic guess was applied to the first 3 carbon neighbors of each adsorption site, and the total initial spin was kept constant during 35 self-consistency cycles (always below 20% of the final number of iterations). A tolerance of 10^{-7} Hartree was set to define the end of the self-consistency, after observing no significant changes from 10^{-9} Hartree in test systems. The Fermi energy is set to zero in all the band structure plots. The resulting atomic magnetic moments are interpolated with a biharmonic spline method[12] as implemented in `MATLAB`.

Mean-field Hubbard model

The conduction band, consisting of p_z electrons, of graphene sheet chemisorbed with hydrogen atoms can be described by the following mean-field Hubbard Hamiltonian[13]

$$\begin{aligned}\mathcal{H} =& t \sum_{<i,j>} \sum_{\sigma} c^{\dagger}_{i,\sigma} c_{j,\sigma} + U \sum_{i} \left[n_{i,\uparrow} \langle n_{i,\downarrow} \rangle + n_{i,\downarrow} \langle n_{i,\uparrow} \rangle \right] \\ &+ \epsilon_H \sum_{i \in \{\text{C-H sites}\}} \sum_{\sigma} n_{i,\sigma} \end{aligned} \tag{1}$$

where $c^{\dagger}_{i,\sigma}$ ($c_{i,\sigma}$) is the creation (annihilation) operator of an electron with spin σ at i^{th} site of the graphene lattice. The first term of the Hamiltonian is the nearest-neighbour hopping term, where parameter t is the hopping parameter and $< i, j >$ signifies pair of nearest-

neighbour sites. The second term is the mean-field Hubbard term describing the on-site Coulomb repulsion between two electrons (which will have opposite spins) present on the same site, where U is the Hubbard potential, $n_{i\sigma} = c^{\dagger}_{i\sigma} c_{i\sigma}$ is the number operator, and $\langle n_{i,\sigma} \rangle$ is the ground state expectation value of the number operator calculated self-consistently. Since the sites on which hydrogen atoms are chemisorbed (C-H sites) are seen as vacancies in the lattice by the p_π electrons, we introduce an on-site potential $\epsilon_H = U/2 + 100t \gg 6t$ (the bandwidth for pristine graphene) through the third term to simulate vacancies.

We take a supercell consisting of N_1 graphene unit cells along its lattice vector $\vec{a}_1 = a(3\hat{x} + \sqrt{3}\hat{y})/2$ and N_2 units cells along $\vec{a}_2 = a(3\hat{x} - \sqrt{3}\hat{y})/2$, where a is the lattice constant of graphene. The lattice vectors of the superlattice are given by $\vec{A}_1 = N_1 \vec{a}_1$ and $\vec{A}_2 = N_2 \vec{a}_2$. For this $N_1 \times N_2$ supercell, we solve the Hamiltonian $\mathcal{H}$ in the Bloch basis, defined as

$$\left|\chi_{\vec{k},\sigma,j}\right\rangle = \frac{1}{\sqrt{N_{s_1} N_{s_2}}} \sum_{\vec{R}} e^{i\vec{k}\cdot\vec{R}} \left|\phi_{\sigma,j,\vec{R}}\right\rangle, \tag{2}$$

where $\left|\phi_{\sigma,j,\vec{R}}\right\rangle$ is the spin σ atomic orbital of the jth site of the supercell situated at the lattice vector $\vec{R}$, $\vec{k}$ is a crystal momentum, and the summation is over all the lattice vectors of the superlattice. N_{s_1} and N_{s_2} are the number of supercells along $\vec{A}_1$ and $\vec{A}_2$, respectively, that consist the superlattice. The real-space projection of the Bloch states satisfy

$$\left\langle \vec{r} + \vec{R} \middle| \chi_{\vec{k},\sigma,j} \right\rangle = e^{i\vec{k}\cdot\vec{R}} \left\langle \vec{r} \middle| \chi_{\vec{k},\sigma,j} \right\rangle, \tag{3}$$

for a lattice vector $\vec{R}$ and a general position vector $\vec{r}$. The Hamiltonian is written as a $4N_1N_2 \times 4N_1N_2$ matrix $\mathcal{H}(\vec{k})$ whose elements are given by

$$\left[\mathcal{H}(\vec{k})\right]_{j\sigma,j'\sigma'} = \left\langle \chi_{\vec{k},\sigma,j} \middle| \mathcal{H} \middle| \chi_{\vec{k},\sigma',j'} \right\rangle = \sum_{\vec{R}''} e^{ik\cdot\vec{R}''} \left\langle \phi_{\sigma,j,\vec{0}} \right| \mathcal{H} \left| \phi_{\sigma',j',\vec{R}''} \right\rangle. \tag{4}$$

Then, $\mathcal{H}(\vec{k})$ is diagonalized

$$\mathcal{H}(\vec{k}) = \sum_m \xi_{\vec{k},m} \left|\xi_{\vec{k},m}\right\rangle \left\langle\xi_{\vec{k},m}\right|, \tag{5}$$

and the local density of states $\rho_{\sigma,j,\vec{0}}(E)$ at energy E for spin σ projected on the jth site of the supercell with lattice vector $\vec{R} = \vec{0}$ is calculated as

$$\rho_{\sigma,j,\vec{0}}(E) = \sum_{\vec{k}} \sum_m \delta(E - \xi_{\vec{k},m}) \left|\left\langle \phi_{\sigma,j,\vec{0}} \middle| \xi_{\vec{k},m}\right\rangle\right|^2, \tag{6}$$

where the Dirac delta function is approximated by

$$\delta(x) = -\frac{1}{\pi} \lim_{\eta \to 0^+} \text{Im}\left(\frac{1}{x + i\eta}\right). \tag{7}$$

Starting from an initial guess value, the local number density $\langle n_{j,\sigma}\rangle$ is calculated self-consistently[14] to obtain its ground state value using the relation

$$\langle n_{j,\sigma}\rangle = \int_{-\infty}^{E_F} \rho_{\sigma,j,\vec{0}}(E)\, dE, \tag{8}$$

where the Fermi energy E_F is set to zero. After reaching the self-consistent solution, energy bands, local spin projection $\langle\sigma_z\rangle = \langle c_\uparrow^\dagger c_\uparrow - c_\downarrow^\dagger c_\downarrow\rangle$, and density of states are calculated.

In all the calculations, supercells were constructed by taking $N_1 = N_2$ and a uniform grid of 144 k-points was used to sample the Brillouin zone by taking $N_{s1} = N_{s2} = 12$. Note that one has to take $U \approx 3.1$ eV and $t \approx 2.5$ eV to get the tight-binding results comparable to the DFT results. In the supporting figures, the parameters used are $U = 3.1$ eV and $t = 2.5$ eV. The calculations presented in Figure 3 in the main text was done on a 30×30 supercell with $U = 5$ eV and $t = 2.5$ eV. We have taken $U = 5$ eV for these calculations so that we get a magnetic solution for the 6 H configuration of Figure 3e (main text). Since the 6 H atoms of Figure 3e are placed very close to each other to fit them in a 30×30 supercell, we get

non-magnetic solution for $U = 3.1$ eV. One would need to take much bigger supercell with larger distances between the H atoms to get a magnetic solution with $U = 3.1$ eV.

Supporting Figures

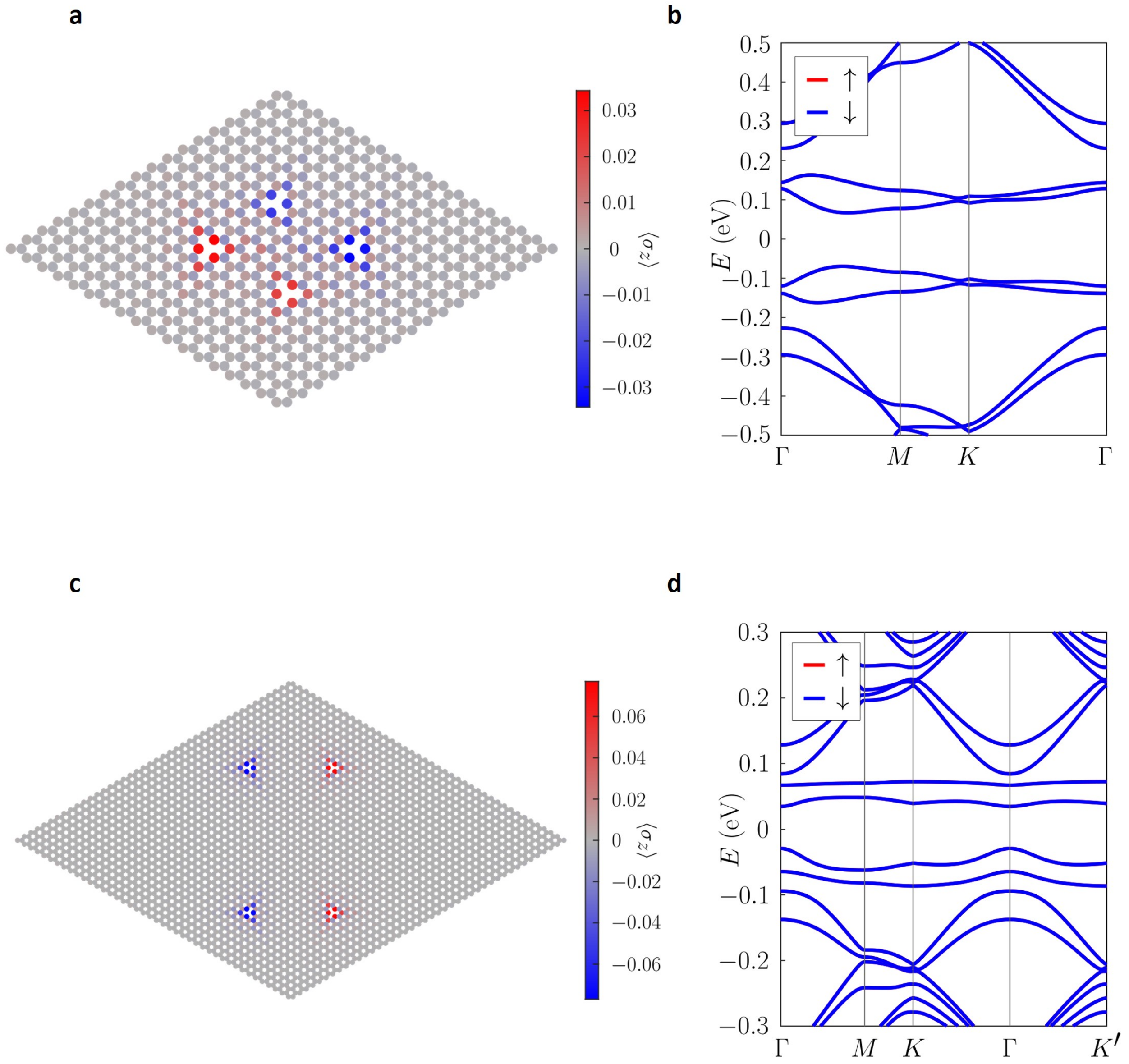

Supporting Figure 1: **Tight-binding calculations of two different 4 H antiferromagnetic configurations** (i.e. with parallelogram symmetry). Magnetization (a,c) and corresponding spin resolved band structure showing Kramer's degeneracy (b,d).

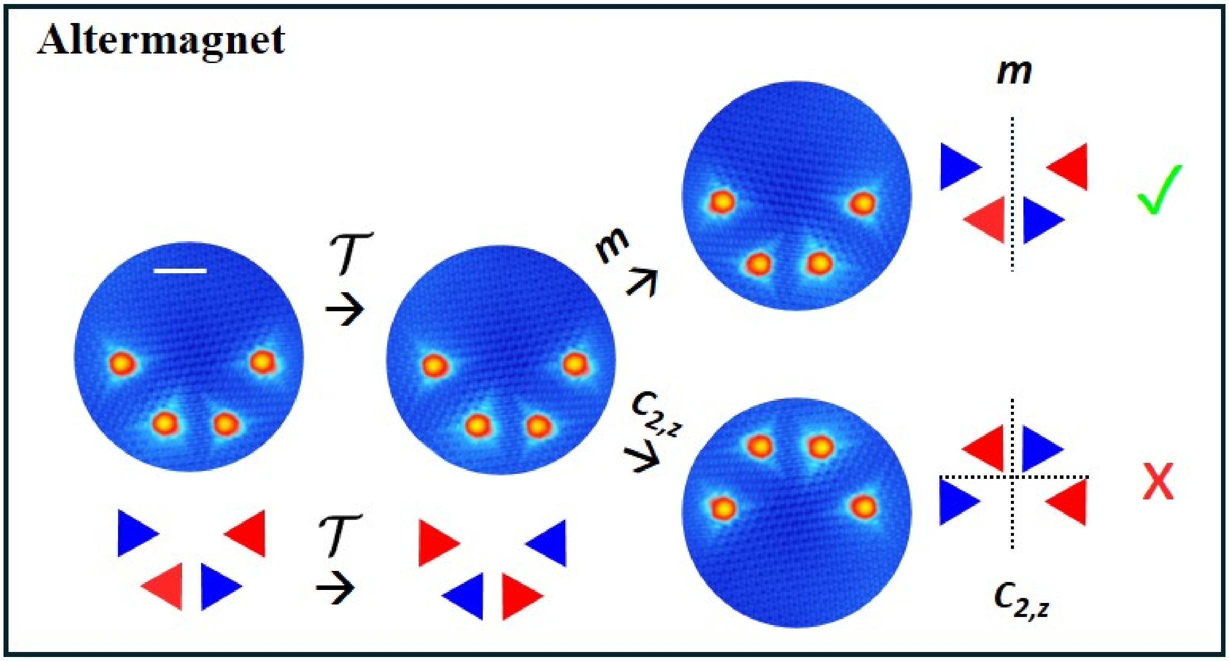

Supporting Figure 2: **Altermagnetic configuration with 4 H atoms** (120mV, 0.1nA, scalebar=2nm). The symmetry requirements are verified on the STM image. After performing a time reversal on spin space ($\mathcal{T}$), a mirror plane (m) recovers the original configuration, while $C_{2,z}$ is broken.

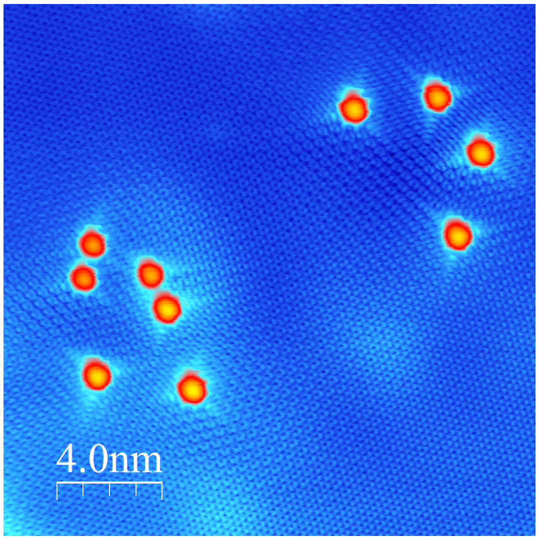

Supporting Figure 3: **Large-scale STM image (50mV, 0.1nA, 20x20nm^2) of the region corresponding to the altermagnetic configuration presented in Figure 3**. The presence of additional hydrogen atoms positioned more than 10 nm away induces long-range electronic perturbations within the graphene lattice, slightly modifying the characteristic pattern surrounding the altermagnetic configuration. These minor perturbations do not compromise the intrinsic symmetry or affect the theoretical predictions associated with the altermagnetic configuration.

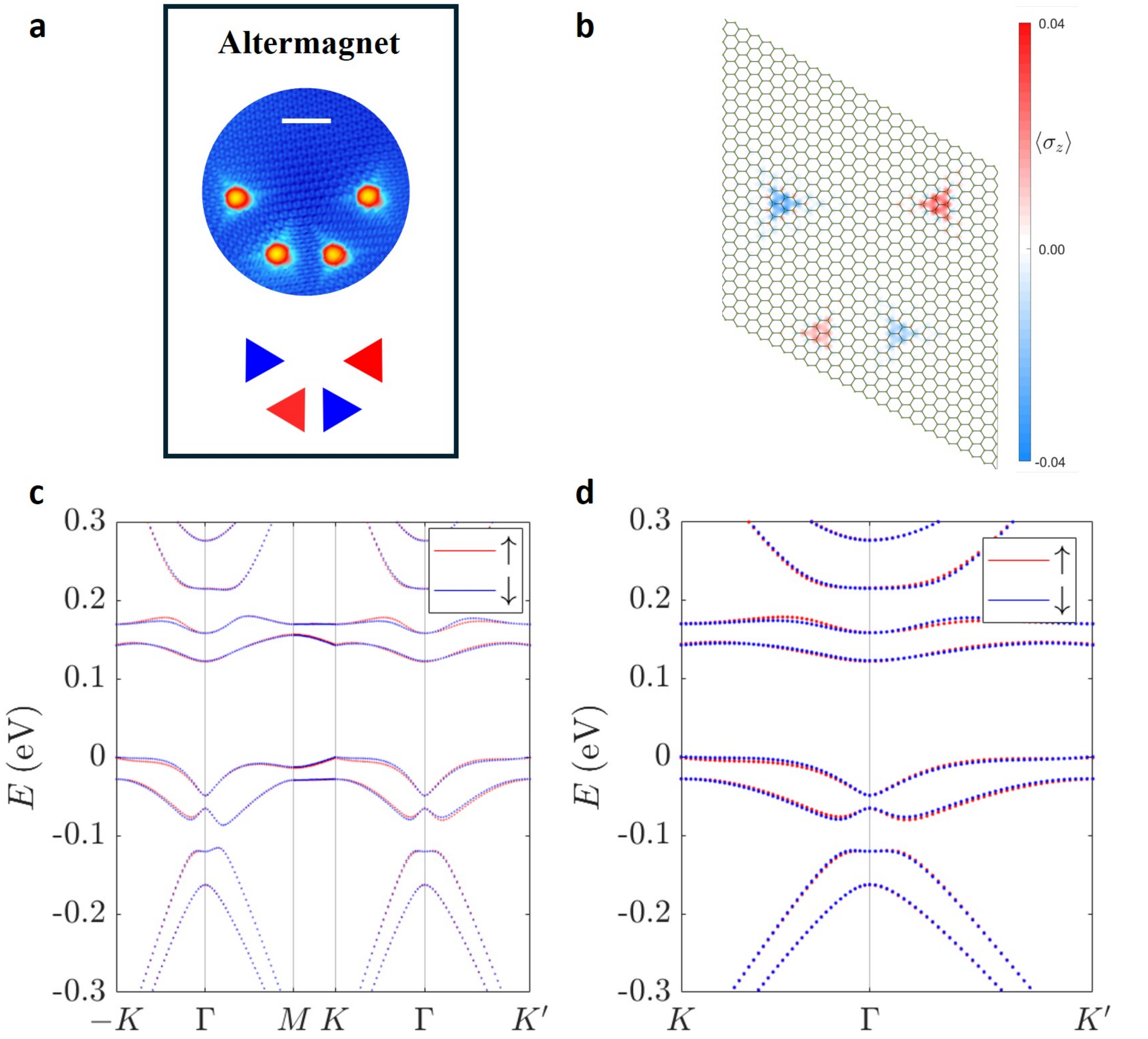

Supporting Figure 4: **DFT calculation of 4 H graphene as an altermagnet.** a) Experimental configuration and symmetry schematics of a 4 H arrangement in graphene with spin group containing a single vertical mirror plane. b) DFT calculated magnetization of a similar altermagnetic configuration. c) Corresponding energy bands d) Zoom of the band structure. STM image: 120mV, 0.1nA, scalebar=2nm.

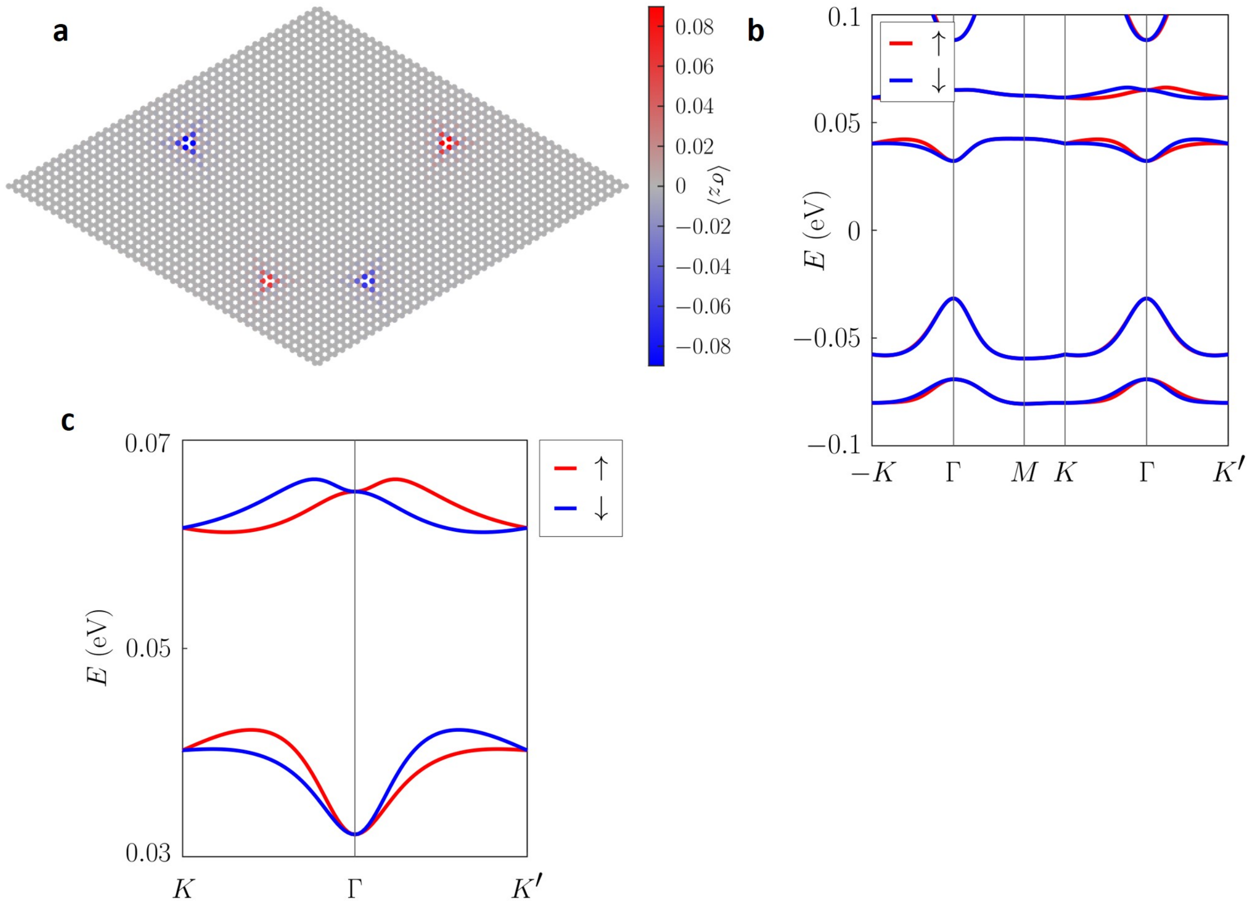

Supporting Figure 5: **Tight-binding calculation of a different 4 H altermagnet on graphene.** Magnetization (a) and energy bands (b and zoom in c) for a different 4 H arrangement in graphene with mirror symmetry and, again, altermagnetic properties.

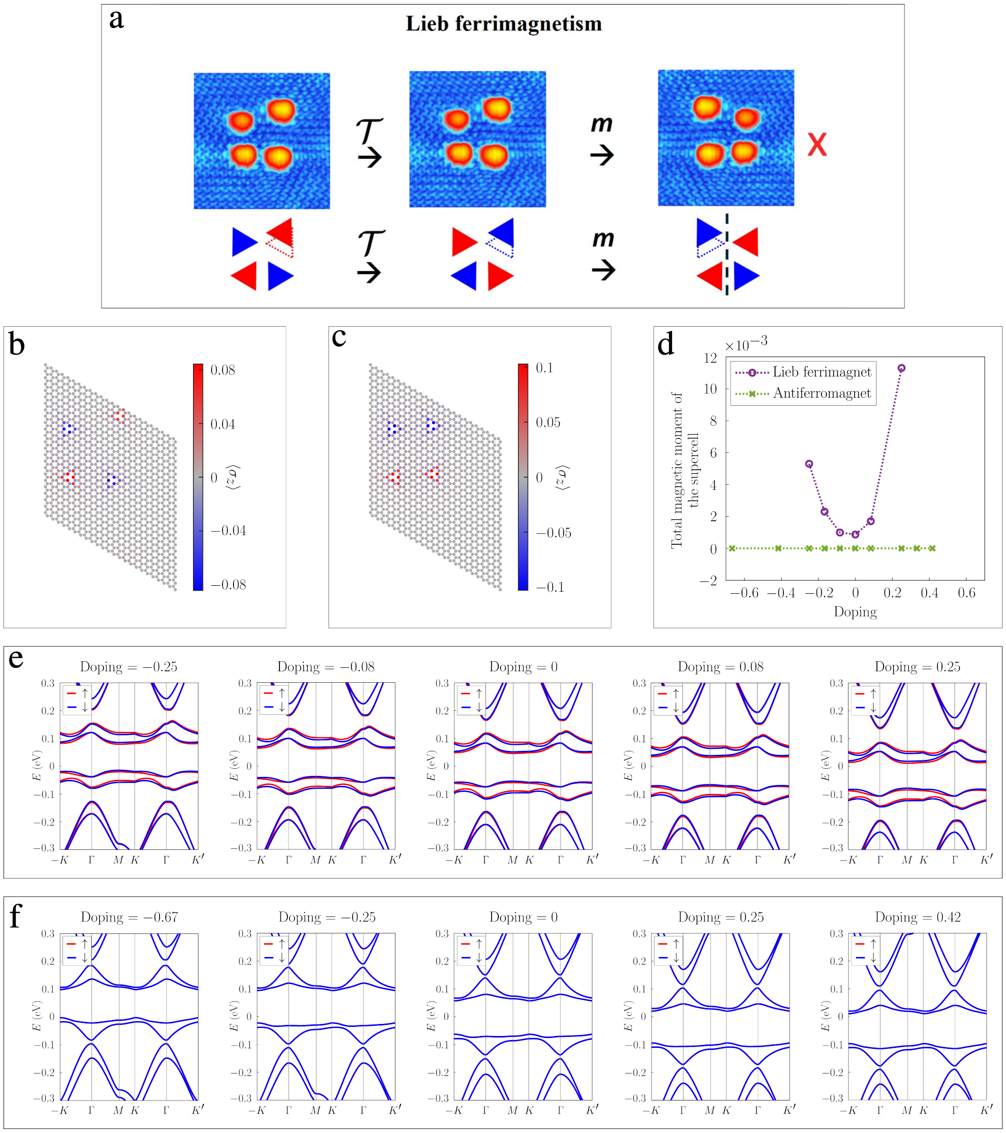

Supporting Figure 6: **Electron doping in a Lieb ferrimagnet.** a) Top, STM image and symmetry transformations applied to the minimal experimental realization of a Lieb ferrimagnet. Bottom, triangle schematics and symmetry transformations. Dashed triangles highlight how crystal symmetry is broken. After $\mathcal{T}$ in spin space, there is no crystal operation that recovers the initial configuration. b) Tight binding magnetization of the broken symmetry configuration shown in a) Top Left with vanishing net magnetization. c) Tight binding magnetization of a centrosymmetric antiferromagnet obtained by rearranging two of the H atoms of configuration b). d) Dependence of the total magnetic moment of the supercell, $\sum_i [\langle n_{i\uparrow}\rangle - \langle n_{i\downarrow}\rangle]/2$, on doping (number of electrons added to the half-filled state of the supercell) for the Lieb ferrimagnet in b) and the antiferromagnet in c). For small doping values, the self-consistency field converges easily and the band structure remains intact. e) Spin resolved electronic band structure of the Lieb ferrimagnet in b) obtained at different doping values. f) Spin-degenerate band structure of the antiferromagnet in c) obtained at different doping values.

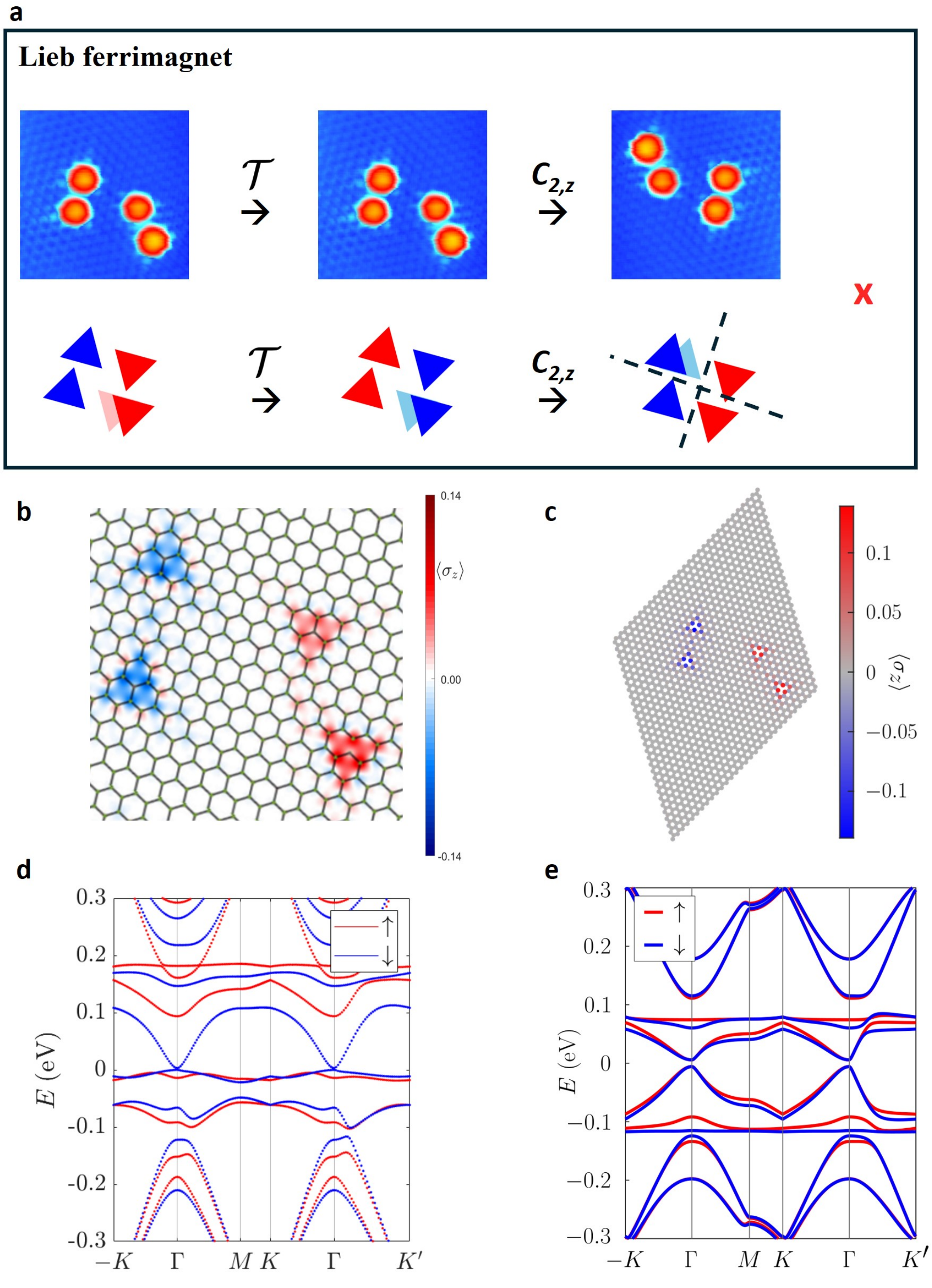

Supporting Figure 7: **Experiment and theory of another 4 H Lieb ferrimagnet on graphene.** a) STM image (100mV, 0.1nA, 6.8x6.8nm^2) and symmetry transformations applied to a different experimental realization of a Lieb ferrimagnet. b,c) Calculated magnetization by DFT (for a scaled down version) and tight binding (for exact scaling as the STM image) respectively. d,e) Spin resolved electronic bands calculated by DFT and tight binding respectively.

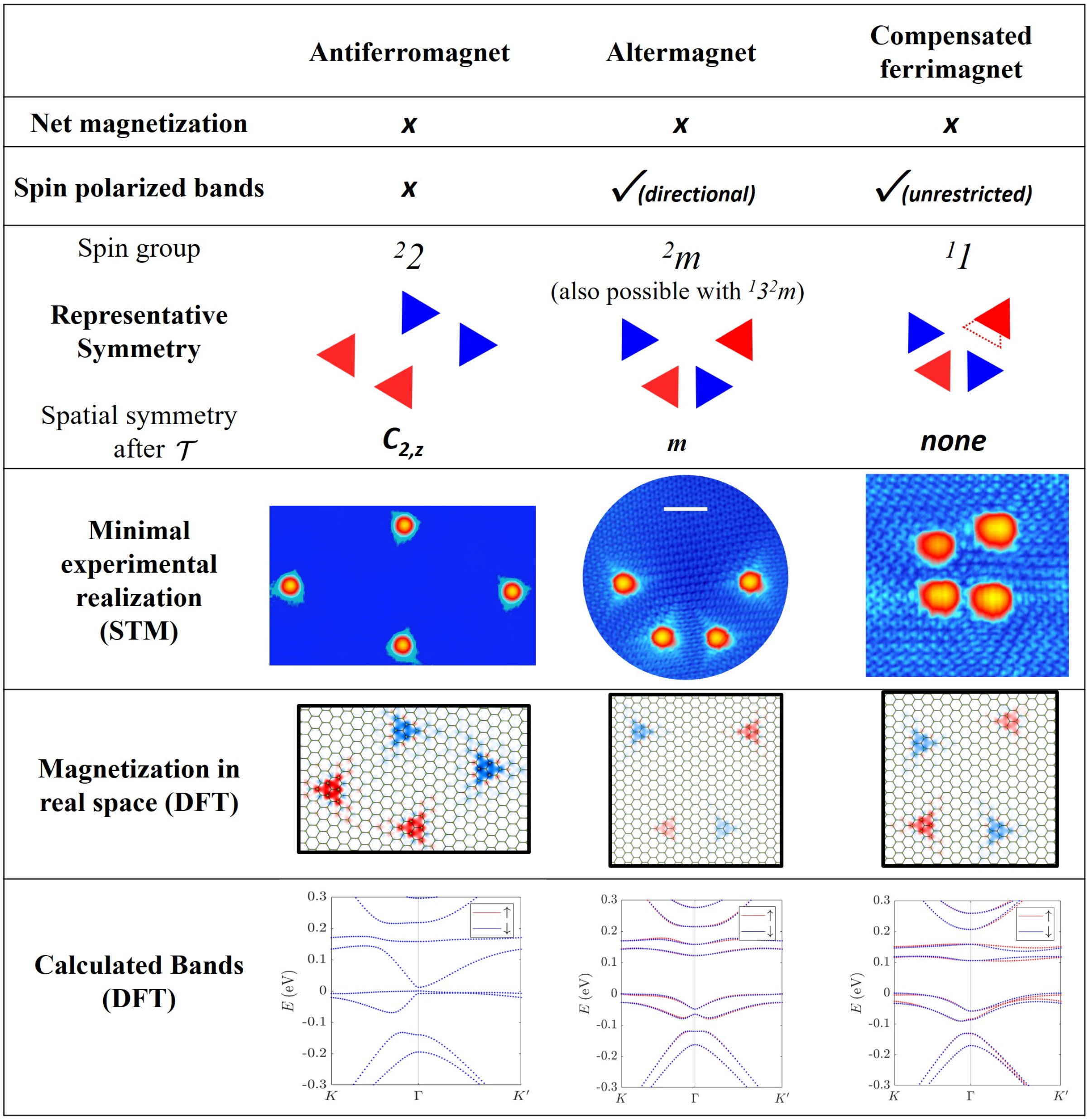

Supporting Figure 8: **Building unconventional magnetic phases with 4 H atoms in graphene.** Summary of all possible collinear, non-relativistic, magnetic phases that can be built on graphene with 4 H atoms and zero net magnetization (even sublattice distribution). By selectively adjusting or breaking the symmetry of the configuration, it is possible to obtain spin polarized bands, in coexistence with a zero net magnetization enforced by Lieb's theorem. The fourth and fifth rows show the STM image and DFT calculated magnetization of each magnetic phase. The last row shows the spin resolved electronic bands. Configurations that fulfill $\mathcal{T} + C_{2,z}$ symmetry correspond to an antiferromagnet, rendering Kramer's degenerate bands; those with $\mathcal{T} + m$ to a d-wave altermagnet, with directional splitting of the bands; and $\mathcal{T}$ broken configurations correspond to fully compensated ferrimagnets, with fully spin-split bands. An alternative i-wave altermagnetic state can also be realized employing 6 H atoms. STM image parameters by columns: (80mV, 0.1nA, 16x25nm^2); (120mV, 0.1nA, scalebar=2nm) ; (30mV, 0.1nA, 7x7nm^2).

References

1. Šmejkal, L.; Sinova, J.; Jungwirth, T. Beyond Conventional Ferromagnetism and Antiferromagnetism: A Phase with Nonrelativistic Spin and Crystal Rotation Symmetry. *Phys. Rev. X* **2022**, *12*, 031042.

2. Varchon, F.; Mallet, P.; Magaud, L.; Veuillen, J.-Y. Rotational disorder in few-layer graphene films on $6H-$SiC(000−1): A scanning tunneling microscopy study. *Phys. Rev. B* **2008**, *77*, 165415.

3. Hass, J.; Varchon, F.; Millán-Otoya, J. E.; Sprinkle, M.; Sharma, N.; de Heer, W. A.; Berger, C.; First, P. N.; Magaud, L.; Conrad, E. H. Why Multilayer Graphene on $4H-$SiC($000\bar{1}$) Behaves Like a Single Sheet of Graphene. *Phys. Rev. Lett.* **2008**, *100*, 125504.

4. González-Herrero, H.; Gómez-Rodríguez, J. M.; Mallet, P.; Moaied, M.; Palacios, J. J.; Salgado, C.; Ugeda, M. M.; Veuillen, J. Y.; Yndurain, F.; Brihuega, I. Atomic-scale control of graphene magnetism by using hydrogen atoms. *Science* **2016**, *352*, 437–441.

5. Hornekær, L.; Rauls, E.; Xu, W.; Šljivančanin, Ž.; Otero, R.; Stensgaard, I.; Lægsgaard, E.; Hammer, B.; Besenbacher, F. Clustering of chemisorbed H(D) atoms on the graphite (0001) surface due to preferential sticking. *Phys. Rev. Lett.* **2006**, *97*, 186102.

6. Horcas, I.; Fernández, R.; Gómez-Rodríguez, J. M.; Colchero, J.; Gómez-Herrero, J.; Baro, A. M. WSXM: A software for scanning probe microscopy and a tool for nanotechnology. *Rev. Sci. Instrum.* **2007**, *78*, 013705.

7. Erba, A.; others CRYSTAL23: a program for computational solid state physics and chemistry. *J. Chem. Theory Comput.* **2022**, *19*, 6891.

8. Perdew, J. P.; Burke, K.; Ernzerhof, M. Generalized gradient approximation made simple. *Phys. Rev. Lett.* **1996**, *77*, 3865.

9. Oliveira, D. V.; Laun, J.; Peintinger, M. F.; Bredow, T. BSSE-correction scheme for consistent gaussian basis sets of double-and triple-zeta valence with polarization quality for solid-state calculations. *J. Comput. Chem.* **2019**, *40*, 2364.

10. Peintinger, M. F.; Oliveira, D. V.; Bredow, T. Consistent Gaussian basis sets of triple-zeta valence with polarization quality for solid-state calculations. *J. Comput. Chem.* **2013**, *34*, 451.

11. Dovesi, R.; others *CRYSTAL23 User's Manual*; University of Torino, 2023.

12. Sandwell, D. T. Biharmonic spline interpolation of GEOS-3 and SEASAT altimeter data. *Geophys. Res. Lett.* **1987**, *14*, 139.

13. Palacios, J. J.; Fernández-Rossier, J.; Brey, L. Vacancy-induced magnetism in graphene and graphene ribbons. *Phys. Rev. B* **2008**, *77*, 195428.

14. Banerjee, A. S.; Suryanarayana, P.; Pask, J. E. Periodic Pulay method for robust and efficient convergence acceleration of self-consistent field iterations. *Chem. Phys. Lett.* **2016**, *647*, 31–35.
